# Supplementary material for: Reanalysis of Published Histological Data Can Help to Characterize Neuronal Death After Spinal Cord Injury
Source: Int J Mol Sci. 2025 Apr 16;26(8):3749. doi: 10.3390/ijms26083749 (PMC12028015; doi:10.3390/ijms26083749)
Supplement: Supplementary file 1 [file ijms-26-03749-s001.zip › ijms-3544453-supplementary/Amezcua et al. Suppl Methods, Figure and Tables.pdf]

SUPPLEMENTARY MATERIALS

Supplementary Figure S1

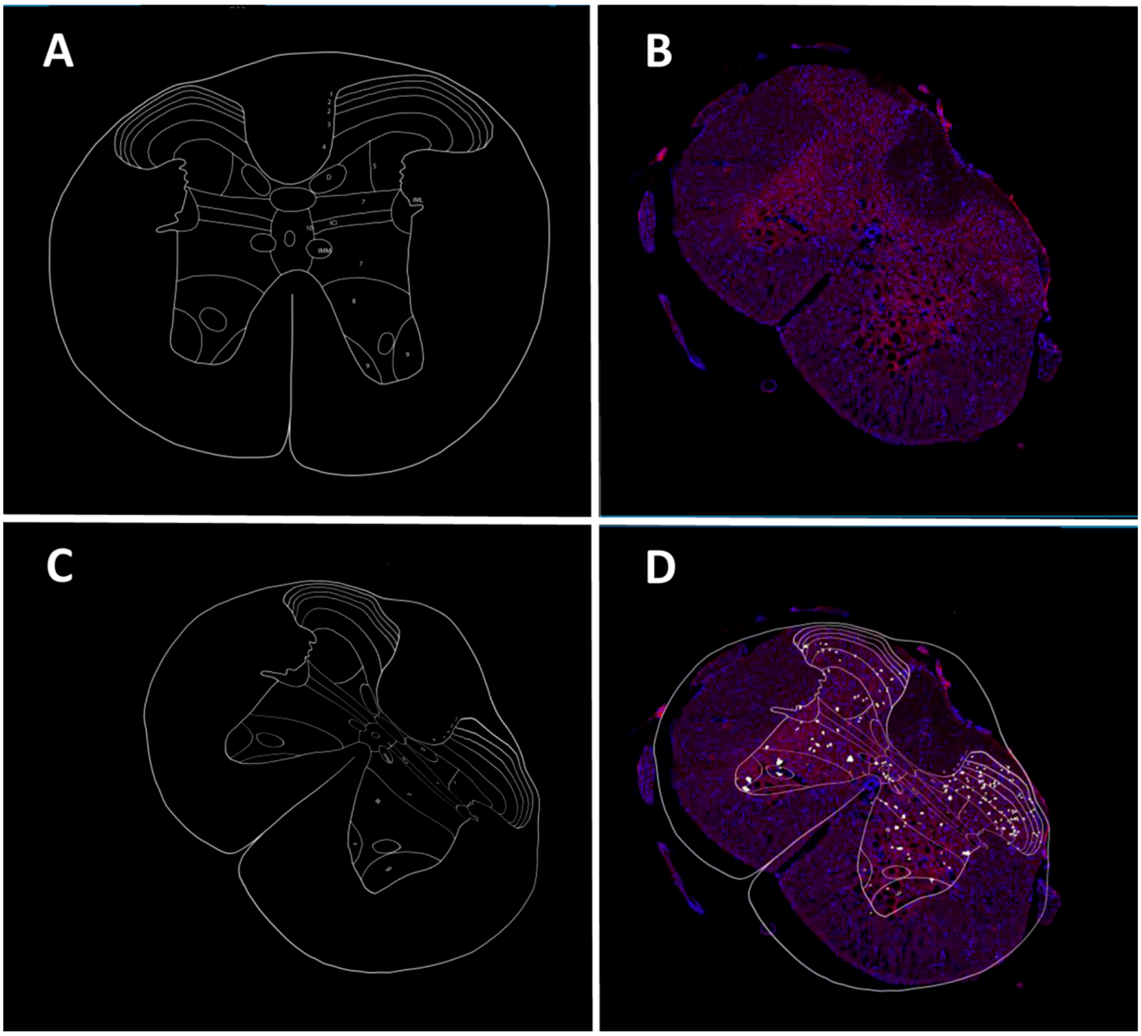

Figure S1. Image registration. A) Atlas of mice T11 transversal section employed as moving image (modified from the mouse spinal cord atlas of Watson *et al.*, 2009), B) Stained image employed as target section. This image comprises DAPI and NeuN layers plus the neural network-based identifications. C) Deformation of the moving image (atlas) to match the stained section form D) Deformations were focused on matching the gray matter contour, without adjusting for the spinal cord contour.

Supplementary Table S1

Image	Condition	Observer						Nº of analyses
		OB180	OB132	OB141	OB109	OB168	OB184	
175.tif	ucf-101		2				1	3
176.tif	ucf-101				2		1	3
177.tif	ucf-101	2		2			1	5
178.tif	ucf-101	2				2	1	5
179.tif	ucf-101				2	2	1	5
180.tif	ucf-101		2		2		1	5
181.tif	ucf-101			2			1	3
182.tif	ucf-101					2	1	3
183.tif	ucf-101	2	2				1	5
184.tif	Vehicle			2	2		1	5
185.tif	Vehicle	2			2		1	5
186.tif	Vehicle		2	2			1	5
187.tif	Vehicle					2	1	3
188.tif	Vehicle			2		2	1	5
189.tif	Vehicle		2				1	3
190.tif	Vehicle	2					1	3
191.tif	Control	2					1	3
193.tif	Control			2			1	3
195.tif	Control		2			2	1	5
197.tif	Control				2		1	3

Table S1. Design for the analysis of the manual identification of neurons. The table details for each image, its code, the type of treatment, the observers who analyzed it, the number of replications performed by each observer, and the total number of analyses performed on each spine section. The observers are indicated by a code (OB + random number) to preserve their anonymity.

Supplementary Table S2

Image	Condition	Number of analysis	Overlap				
			1	2	3	4	5
175.tif	Ucf-101	3	105	104	283		
176.tif	Ucf-101	3	298	106	87		
177.tif	Ucf-101	5	56	42	46	67	218
178.tif	Ucf-101	5	78	44	64	72	230
179.tif	Ucf-101	5	43	20	37	58	131
180.tif	Ucf-101	5	87	20	7	4	11
181.tif	Ucf-101	3	40	25	29		
182.tif	Ucf-101	3	55	49	159		
183.tif	Vehicle	5	82	32	19	19	7
184.tif	Vehicle	5	198	95	16	1	0
185.tif	Vehicle	5	30	11	0	0	0
186.tif	Vehicle	3	40	11	0	0	0
187.tif	Vehicle	3	15	5	0		
188.tif	Vehicle	5	93	24	14	6	3
189.tif	Vehicle	3	263	89	85		
190.tif	Vehicle	3	37	7	3		
191.tif	Control	3	171	161	216		
193.tif	Control	3	163	150	427		
195.tif	Control	5	167	106	110	105	196
197.tif	Control	3	228	124	224		

Table S2. Coherence in manual identifications. The table shows the results obtained after overlapping the manual identifications for each image of the 20 spinal cord sections. The table details the code of each image/section, the condition of the individual, the total number of analyses performed on each section, whereas overlap describes the number of neurons with overlapped counts in each section. The value of 1 refers to neurons that have only been identified in one analysis, the value of 2 corresponds to neurons identified in two analyses, and so on until the values of 5.

Supplementary Table S3

Imag e	Condition	Analysis	Number of counts						
			0	1	2	3	4	5	Total
178.tif	ucf-101	Manual		78	44	64	72	230	488
		Threshold +	36	14	8	19	30	169	276
		NeurNet +	64	63	41	55	70	228	521
180.tif	ucf-101	Manual		87	20	7	4	11	129
		Threshold +	84	11	7	7	2	10	121
		NeurNet +	31	10	7	6	3	11	68
183.tif	Vehicle	Manual		82	32	19	19	7	159
		Threshold +	224	29	20	15	11	7	306
		NeurNet +	43	18	16	12	13	7	109
188.tif	Vehicle	Manual		93	24	14	6	3	140
		Threshold +	250	61	18	10	6	3	348
		NeurNet +	35	13	9	7	4	3	71
195.tif	Control	Manual		167	106	110	105	196	684
		Threshold +	156	47	51	77	82	176	589
		NeurNet +	44	80	74	89	87	191	565

Table S3. Agreement among neuronal identifications. The table shows the agreement between neuronal identifications obtained using manual, threshold-based, and neural network-based (NeurNet) methods. The table details the code of each image (Image), the condition (Treatment), the employed identification method (Analysis), and the number of identified neurons (Number of counts). The Number of counts is split into 6 classes according to the number of objects identified as neurons in 0 to 5 manual analyses. Classes 1 to 5 refer to the objects identified as neurons in one, two, three, four, or five of the manual analyses, respectively. The value zero corresponds to objects identified as neurons only by threshold or neural network-based methods. “Threshold +” and “NeuroNet +” indicate the number of objects identified as neurons in each class also identified by these methods.

Supplementary Table S4

Image	RNN	Manual analyses						Threshold	NC-AI1
		OBS109	OBS132	OBS141	OBS168	OBS180	OBS184		
175.tif	386		377				431	116	311
176.tif	255	170					244	88	305
177.tif	327			346.5		363	327	76	416
178.tif	359				357.5	371.5	352	81	466
179.tif	216	202.5			240		222	117	331
180.tif	44	35.5	95				21	19	42
181.tif	59			82.5			71	74	117
182.tif	209				218.5		224	55	275
183.tif	63		79.5			59.5	51	3	103
184.tif	88	28		168			179	3	133
185.tif	10	22.5				1	6	16	18
186.tif	12		49	0			0	2	0
187.tif	5				13.5		0	13	0
188.tif	44			93.5	42		15	4	51
189.tif	231		301				145	45	118
190.tif	20					23.5	16	13	35
191.tif	379					320.5	507	142	260
193.tif	580			597			605	168	590
195.tif	422		481.5		403		542	114	505
197.tif	381	301					568	108	548

Table S4. Total number of neurons in each section. The table shows the Reference Number of Neurons (RNN) as well as the estimates from the manual, threshold and neural network-based methods for each image under analysis. The code OB168, etc. anonymously identifies the analyst involved.

Supplementary Table S5

Condition	Control	Vehicle				Ucf-101			
D.T.E. (mm)	-	0.6	0.8	1.0	1.2	0.6	0.8	1.0	1.2
N. of images	4	3	5	6	4	5	5	4	1

Table S5. Number of images (sections) analyzed in each condition and distance to the epicenter. Additional data on the samples are available at https://osf.io/bhg6f. D.T.E. indicates distance to epicenter

Supplementary Table S6

		L1	L2	L2I	L3	L4	L5I	L5m	D	L7	ICI	IML	L7B	IMM	L8	L9	L10	LDC	Total
Control	med	25.8	23.0	21.8	50.0	37.0	20.3	8.0	4.0	10.5	8.5	3.3	27.5	3.3	16.8	3.5	19.0	10.5	282.5
	%	9.1	8.1	7.7	17.7	13.1	7.2	2.8	1.4	3.7	3.0	1.2	9.7	1.2	5.9	1.2	6.7	3.7	100.0
T11 Atlas	med	15.0	48.0	59.0	126.0	140.0	103.0	24.0	7.0	31.0	28.0	13.0	51.0	4.0	46.0	16.0	37.0	30.0	778.0
	%	1.9	6.2	7.6	16.2	18.0	13.2	3.1	0.9	4.0	3.6	1.7	6.6	0.5	5.9	2.1	4.8	3.9	100.0
ratio %Ctrl/ %Atlas		4.7	1.3	1.0	1.1	0.7	0.5	0.9	1.6	0.9	0.8	0.7	1.5	2.2	1.0	0.6	1.4	1.0	

Table S6. Median number of neurons per Rexed laminae in controls and the Mouse Spinal Cord Atlas from Watson *et al.* (2009). Percentage relative to the total number of neurons in a section is also shown to evaluate the agreement between data from the Watson *et al.* (2009) reference atlas and our results. The last row shows the ratio between the percentages of neurons in each laminae estimated from the Watson *et al*. (2009) atlas and our data. Major changes (above 2 fold) are shown in red type.

Supplementary Table S7

Condition	DTE	N	L1	L2	L2b	L3	L4	L5l	L5m	D	L7	ICl	IML	L7b	IMM	L8	L9	L10	LDCom	Total
Control		3	25.8	23.0	21.8	50.0	37.0	20.3	8.0	4.0	10.5	8.5	3.3	27.5	3.3	16.8	3.5	19.0	10.5	282.5
Vehicle	0.6	3	0**	1	0*	1.5*	2	0	0	0	0	0	0	2*	0*	1*	0*	0	0*	6.5*
	0.8	5	2*	5	7*	15.5*	10	4	1	0	2	1*	1	5.25*	0**	4*	1	2	0**	62*
	1.0	6	9	7	9	20	14	6	2	1	3	2	3	12	1	6	2	5	2	112
	1.2	4	9	9	10	18	10	5	2	1	7	3	3	13	2	8	3	6	4	112
UCF	0.6	5	5**	6	2*	8*	7	3	1	0.5*	2	1	0.5**	5**	1	2*	1	0	2	45**
	0.8	5	6.5*	7	6	10	14	10	1	2	5	2	2	6	0**	5	1	5	1#	80
	1.0	4	9	7	10	18	21	10	3	2	5	3	1	9	1	4	2	4	2	110
	1.2	1	16	7	7	14	19	18	4	4	6	2	3	5	1	7	4	2	3	119

Table S7. Number of neurons in sections from un-damaged spinal cords and from damaged ones sampled 21 days after injury at 0.6, 0.8, 1.0, and 1.2 mm caudal to the contusion epicenter. The table details the median values of the number of neurons in each section and in each laminae and nucleus of the T11 spinal cord segment before and after SCI with or without ucf–101 treatment. For vehicle and ucf-101 data, statistically significant neuronal losses relative to control values after Kruskal Wallis test and one-tailed Dunn's posthoc test are marked for each region and distance to the epicenter. Protection due to ucf-101 (reduction in neuronal losses) is estimated by comparing ucf-1001 data at each laminae and distance with the corresponding data from the vehicle-treated individuals using Wilcoxon rank-sum test. " * " and " ** " indicates *p*<0.05 and *p*<0.01 significance after one-tailed Dunn's posthoc test, "#" indicates p<0.05 significance after Wilcoxon rank-sum test. Statistical analyses are available in the OSF project (https://osf.io/awkgu). DTE indicates Distance to Epicenter, expressed in mm. N indicates the number of individuals.
